# Anxiety and Depression in Heart Failure

**DOI:** 10.19102/icrm.2025.16114

**Published:** 2025-11-15

**Authors:** Maria Polikandrioti, Athanasia Tsami

**Affiliations:** 1 Nursing Department, University of West Attica, Athens, Greece; 2 PhD Candidate, Nursing Department, University of West Attica, Athens, Greece

**Keywords:** Anxiety, depression, heart failure, Hospital Anxiety and Depression Scale

## Abstract

Patients with heart failure (HF) may experience depression or anxiety due to various reasons associated with their or caregivers’ characteristics. The purpose of this study was to explore patients’ and caregivers’ characteristics associated with hospitalized HF patients’ anxiety and depression. A total of 300 hospitalized HF patients with their caregivers were enrolled in the study. Data were collected using the Hospital Anxiety and Depression Scale, which also included patients’ and caregivers’ characteristics. The statistical significance level was set at *P* < .05. A statistically significant association was observed between patients’ anxiety and age (*P* = .044), level of education (*P* = .015), type of diagnosis (*P* = .001), New York Heart Association (NYHA) class (*P* = .001), prior hospitalization within the current year (*P* = .013), current smoking (*P* = .001), frequency of physical exercise (*P* = .001), and their self-reported ability for symptom management after hospital discharge (*P* = .001). A statistically significant association was observed between patients’ depression and age (*P* = .018), type of diagnosis (*P* = .001), NYHA class (*P* = .001), prior hospitalization within the current year (*P* = .004), current smoking (*P* = .001), occasional alcohol consumption (*P* = .026), frequency of physical exercise (*P* = .001), and their self-reported ability for symptom management after hospital discharge (*P* = .001). In terms of caregivers’ characteristics, a statistically significant association was observed between patients’ anxiety/depression and the relationship with caregivers (*P* = .006 and *P* = .001, respectively), whether caregivers declared added responsibilities among family members (*P* = .041 and *P* = .002, respectively), and whether they felt uncertain about patients’ clinical outcome (*P* = .001 and *P* = .001, respectively). Finally, a statistically significant association was observed between patients’ depression and the occupation of their caregivers (*P* = .038). Patients’ characteristics associated with anxiety/depression were demographic and clinical, while caregivers’ characteristics associated with patients’ anxiety/depression were their self-reports and demographic characteristics. Knowledge of factors that influence anxiety and depression can enable health care professionals to offer appropriate interventions tailored to their needs.

## Introduction

Patients with heart failure (HF) experience anxiety and depression, which adversely affect their lives. According to estimates in HF patients, the prevalence of anxiety ranges from 20%–50% and that of depression ranges from 20%–45%, highlighting their psychological burden.^1^

Anxiety and depression in HF patients have negative effects on patients’ outcomes. Anxiety accounts for hospital readmissions in one-third of HF patients.^1,2^ Compared to the general population, depression is approximately five times more common in chronic HF patients and doubles the risk of mortality, regardless of other well-established risk factors, such as age, family history, and social isolation.^2^ Furthermore, moderate-to-severe depression accounts for a two-fold increased risk of hospitalization and visits to the emergency department.^3,4^ The combination of anxiety and depression is a predictor of all-cause mortality in HF patients.^5^

Anxiety and depression are mainly associated with disorders in the autonomic nervous system, immune system, and metabolic health as well as with hypothalamic–pituitary–adrenal axis dysfunction.^6–8^ Anxiety keeps pace with depression, as it often precedes or coexists with depression, with overlapping symptoms.^8^ Additionally, anxiety and depression negatively affect treatment adherence, adoption of necessary lifestyle modifications, and social functioning.^1,2^ Anxiety and depression share a bidirectional relationship with HF. For example, HF exacerbation episodes may trigger the onset of anxiety/depression, while already established anxiety/depression impairs patients’ physical and mental strength, thus entailing frequent hospitalizations or slower recovery.^4^

Hospitalizations are a crucial point on the illness trajectory, as health care professionals are offered the opportunity to evaluate the mental health of HF patients and moreover to schedule their monitoring after hospital discharge, as, during this period, they are more vulnerable to readmission.^9^

HF patients heavily rely on family caregivers for help with daily activities such as daily symptom monitoring, adherence to dietary restrictions, and support for complex medication regimens. Frequently, but not inevitably, the caregivers’ status may be a determinant of patients’ anxiety and depression.^10^ Efforts are needed to explore patient and caregiver factors associated with anxiety and depression in the long-term management of HF. In clinical settings, information regarding caregivers’ characteristics always needs to be included in the assessment of HF complexity.

In an attempt to address all these issues, this study aimed to explore HF patients’ and caregivers’ characteristics associated with patients’ anxiety and depression.

## Materials and methods

### Design, setting, and period of the study

In this cross-sectional study, the sample consisted of 300 HF patients hospitalized in a public hospital from 2022–2024 and their family caregivers. The dyad (patient, caregiver) was selected using convenience sampling.

### Inclusion and exclusion criteria of the sample

The inclusion criteria for patients and caregivers were as follows: (1) ability to comprehend the Greek language and (2) ability to read and sign the informed consent form. For patients, the inclusion criterion was hospitalization solely for HF.

### Data collection and procedure

Data collection was performed through interviews, which lasted approximately 30 min. The interview took place during evening shifts when both patients and caregivers were available.

### Research instrument

Data were collected using the Hospital Anxiety and Depression Scale (HADS), and patient and caregiver characteristics were also recorded. The demographic characteristics of patients included age, sex, education, and occupation. Patients’ clinical characteristics included recorded diagnosis, New York Heart Association (NYHA) functional classification, prior hospitalization within the current year, occasional alcohol consumption, coffee consumption, current smoking, frequency of exercise, coronavirus disease 2019 (COVID-19) vaccination, and self-reported ability for symptom management after hospital discharge.

In terms of caregivers’ characteristics, the following were recorded: their relation to the patient, sex, age, education, occupation, frequency of hospital visits, level of information about HF, whether they felt uncertain about patients’ clinical outcome (ie, the change in the patient’s health resulting from care and treatment), whether they believed that caregivers need support, whether they neglected their personal life, and whether they declared added responsibilities within family members.

### Measurement of anxiety and depression

For the evaluation of the mental health (depression and anxiety) of the patients, HADS was used. This scale, which was proposed in 1983 by Zigmond and Snaith,^11^ consists of 14 questions that assess how patients felt during the previous week. Patients were able to answer every question on a 4-point Likert scale of 0–3 points. Of the 14 questions, seven assess depression and the other seven assess anxiety. Scores attributed to questions are summed separately for anxiety and depression, leading to two scores ranging from 0–21 points. Higher scores indicate greater levels of anxiety and depression.^11^ HADS has been tested for its validity and reliability in the Greek population by Michopoulos et al.,^12^ where a score of >8 points indicates anxiety and depression.

### Ethical considerations

This study was approved by the research committee of the public hospital. Participants were informed by the researcher regarding the purposes of the study, and their written informed consent was obtained. Data collection guaranteed anonymity and confidentiality. All subjects were informed of their rights to refuse or discontinue participation in the study, according to the ethical standards of the Declaration of Helsinki (1989) of the World Medical Association.

### Statistical analysis

Categorical data are presented as absolute and relative frequencies (%), while continuous data are presented as mean and standard deviation values. Non-parametric tests such as the Mann–Whitney *U* and Kruskal–Wallis tests were used to test for the association of patients’ and caregivers’ characteristics with patients’ anxiety and depression. Multiple linear regression was performed to estimate the effect of patients’ and caregivers’ characteristics on patients’ anxiety and depression. Results are presented as β regression coefficients and 95% confidence intervals (CIs). The observed level of 5% was considered statistically significant. All statistical analyses were performed using SPSS version 28 (IBM Corp., Armonk, NY, USA).

## Results

### Description of patients’ sample

**Table 1** presents the description of the patients’ sample. The majority of patient-participants were men (65.3%), aged >70 years (55.6%), had only a primary level education (42.7%), were pensioners (72.1%), were diagnosed with HF decompensation (76.3%), were of NYHA class III (44.0%), had prior hospitalization within the current year (74.3%), occasionally consumed alcohol (52.0%), drank coffee (82.3%), never performed any type of exercise (47.3%), and had received a COVID-19 vaccine (75.3%), while less than half of them believed that they were able to manage symptoms after hospital discharge (47.3%).

**Table 1:** Distribution of the Patients’ Sample According to Their Characteristics (n = 300)

	n (%)		n (%)
Sex		HF diagnosis	
Male	196 (65.3%)	Chronic	71 (23.7%)
Female	104 (34.7%)	Decompensated	229 (76.3%)
Age (years)		NYHA class	
51–60	45 (15.0%)	I	11 (3.7%)
61–70	88 (29.3%)	II	97 (32.3%)
71–80	127 (42.3%)	III	132 (44.0%)
>80	40 (13.3%)	IV	60 (20.0%)
Education		Other characteristics	
Primary	128 (42.7%)	COVID-19 vaccination status (vaccinated)	225 (75.3%)
Secondary	100 (33.3%)	Smoker (yes)	80 (26.7%)
University	22 (7.3%)	Alcohol consumption (yes)	156 (52.0%)
MSc, PhD	213 (71.0%)	Coffee consumption (yes)	247 (82.3%)
Occupation		Exercise	
Unemployed	12 (3.5%)	Never	142 (47.3%)
Employed	55 (18.3%)	1–2 times/week	73 (24.3%)
Household	24 (7.0%)	3–4 times/week	28 (9.3%)
Pensioner	245 (72.1%)	>4 times/week	57 (19.0%)
Ability to manage symptoms (yes)	142 (47.3%)	Prior hospitalization within the current year (yes)	223 (74.3%)

*Abbreviations:* COVID-19, coronavirus disease 2019; HF, heart failure; NYHA, New York Heart Association.

### Description of caregivers’ sample

**Table 2** presents the description of the caregivers’ sample. The majority of caregiver-participants were women (69.0%), were spouses (65.0%), aged >60 years (43.0%), had secondary education (39.3%), were employed (51.0%), were enough informed about HF (43.0%), were in the hospital during the whole admission (43.7%), declared they felt uncertain about the patient’s clinical outcome (81.0%), declared added responsibilities within family members (66.0%), believed that caregivers need support (88.6%), and felt they neglected their personal life (75.7%).

**Table 2:** Distribution of the Caregivers’ Sample According to Their Characteristics (n = 300)

	n (%)		n (%)
Relation to patient		Informed about patient’s HF	
Child	105 (35.0%)	Very	119 (39.7%)
Husband/wife	195 (65.0%)	Enough	129 (43.0%)
Gender		A little	52 (17.3%)
Male	93 (31.0%)	Not at all	0 (0.0%)
Female	207 (69.0%)	Frequency of hospital visits	
Age (years)		In hospital the whole admission	131 (43.7%)
<40	47 (15.7%)	Once a day	108 (36.0%)
41–50	65 (21.7%)	Twice a day	18 (6.0%)
51–60	59 (19.7%)	Day by day	41 (13.7%)
61–70	62 (20.7%)	Uncertain about patients’ outcome	
71–80	67 (22.3%)	Very	102 (34.0%)
Education		Enough	141 (47.0%)
Primary	65 (21.7%)	A little	50 (16.7%)
Secondary	118 (39.3%)	Not at all	7 (2.3%)
University	99 (33.0%)	Do caregivers need support?	
MSc, PhD	18 (6.0%)	Very	91 (30.3%)
Occupation		Enough	175 (58.3%)
Unemployed	8 (2.7%)	A little	28 (9.3%)
Employed	153 (51.0%)	Not at all	6 (2.0%)
Household	65 (21.7%)	Do you neglect your personal life when caregiving?	
Pensioner	73 (24.3%)	Very	87 (29.0%)
Added responsibilities among family members? (yes)	198 (66.0%)	Enough	140 (46.7%)
		A little	58 (19.3%)
		Not at all	15 (5.0%)

*Abbreviation:* HF, heart failure.

### Patients’ anxiety and depression

The results showed that 81.0% (n = 243) of the patients had anxiety and 77.0% (n = 231) depression, with a cut-off score of 8 points. Fifty-seven (19.0%) participants had no anxiety (score, ≤8 points) and 69 had no depression (23.0%).

### Factors associated with heart failure patients’ anxiety and depression

**Table 3** presents the association of HF patients’ characteristics with anxiety and depression.

**Table 3:** Patients’ Characteristics Associated with Their Anxiety and Depression (HADS Scores)

Patients’ Characteristics	Patient Anxiety			Patient Depression		
	No, n (%)	Yes, n (%)	*P* Value	No, n (%)	Yes, n (%)	*P* Value
Gender			.586			.756
Male	39 (19.9%)	157 (80.1%)		44 (22.4%)	152 (77.6%)	
Female	18 (17.3%)	86 (82.7%)		25 (24.0%)	79 (76.0%)	
Age (years)			**.044**			**.018**
≤60	13 (28.9%)	32 (71.1%)		15 (33.3%)	30 (66.7%)	
61–70	21 (23.9%)	67 (76.1%)		27 (30.7%)	61 (69.3%)	
71–80	16 (12.6%)	111 (87.4%)		20 (15.7%)	107 (84.3%)	
>80	7 (17.5%)	33 (82.5%)		7 (17.5%)	33 (82.5%)	
Education			**.015**			.122
Primary	16 (12.5%)	112 (87.5%)		23 (18.0%)	105 (82.0%)	
Secondary	20 (20.0%)	80 (80.0%)		24 (24.0%)	76 (76.0%)	
University/MSc/PhD	21 (29.2%)	51 (70.8%)		22 (30.6%)	50 (69.4%)	
Occupation			.217			.186
Unemployed/household	6 (18.8%)	26 (81.3%)		9 (28.1%)	23 (71.9%)	
Employee	15 (27.3%)	40 (72.7%)		17 (30.9%)	38 (69.1%)	
Pensioner	36 (16.9%)	177 (83.1%)		43 (20.2%)	170 (79.8%)	
HF diagnosis			**.001**			**.001**
Chronic	29 (40.8%)	42 (59.2%)		32 (45.1%)	39 (54.9%)	
Decompensation	28 (12.2%)	201 (87.8%)		37 (16.2%)	192 (83.8%)	
NYHA class			**.001**			**.001**
I–II	38 (35.2%)	70 (64.8%)		46 (42.6%)	62 (57.4%)	
III	19 (14.4%)	113 (85.6%)		21 (15.9%)	111 (84.1%)	
IV	0 (0.0%)	60 (100.0%)		2 (3.3%)	58 (96.7%)	
Vaccinated against COVID-19			.518			.353
Yes	41 (18.2%)	184 (81.8%)		49 (21.8%)	176 (78.2%)	
No	16 (21.6%)	58 (78.4%)		20 (27.0%)	54 (73.0%)	
Smoker			**.001**			**.001**
Yes	25 (31.3%)	55 (68.8%)		31 (38.8%)	49 (61.3%)	
No	32 (14.5%)	188 (85.5%)		38 (17.3%)	182 (82.7%)	
Coffee consumption			.424			.431
Yes	49 (19.8%)	198 (80.2%)		59 (23.9%)	188 (76.1%)	
No	8 (15.1%)	45 (84.9%)		10 (18.9%)	43 (81.1%)	
Alcohol consumption			.061			**.026**
Yes	36 (23.1%)	120 (76.9%)		44 (28.2%)	112 (71.8%)	
No	21 (14.6%)	123 (85.4%)		25 (17.4%)	119 (82.6%)	
Exercise			**.001**			**.001**
Never	17 (12.0%)	125 (88.0%)		19 (13.4%)	123 (86.6%)	
1–2 times/week	12 (16.4%)	61 (83.6%)		16 (21.9%)	57 (78.1%)	
>2 times/week	28 (32.9%)	57 (67.1%)		34 (40.0%)	51 (60.0%)	
Prior hospitalization			**.013**			**.004**
Yes	35 (15.7%)	188 (84.3%)		42 (18.8%)	181 (81.2%)	
No	22 (28.6%)	55 (71.4%)		27 (35.1%)	50 (64.9%)	
Ability to manage symptoms			**.001**			**.001**
Yes	39 (27.5%)	103 (72.5%)		51 (35.9%)	91 (64.1%)	
No	18 (11.4%)	140 (88.6%)		18 (11.4%)	140 (88.6%)	

*Abbreviations:* COVID-19, coronavirus disease 2019; HADS, Hospital Anxiety and Depression Scale; HF, heart failure; NYHA, New York Heart Association. Bold *P*-values are statistically significant.

A statistically significant correlation was observed between patients’ anxiety and age (*P* = .044), level of education (*P* = .015), type of diagnosis (*P* = .001), NYHA class (*P* = .001), prior hospitalization within the current year (*P* = .013), whether they were current smokers (*P* = .001), frequency of physical exercise (*P* = .001), and their self-reported ability for symptom management after hospital discharge (*P* = .001). More specifically, patients <60 years of age had a lower rate of anxiety (71.1%) compared to the other age groups, especially the age group 71–80 years, which presented an anxiety rate of 87.4%. The higher the age group, the greater the anxiety rate. Patients with only a primary education had a higher rate of anxiety (87.5%) compared to those with higher education (secondary, 80.0%; university, 70.8%). Similarly, a higher rate of anxiety was observed in HF patients with decompensation (87.8%) compared to those with chronic HF (59.2%). Likewise, a higher rate of anxiety was found in patients with NYHA class IV (100.0%) compared to those with NYHA class III (85.6%) and NYHA class I–II (64.8%), those who had prior hospitalization within the current year (84.3%), those who did not smoke (85.5%), those who never performed any type of exercise (88.0%), and those who declared not to have the ability for symptom management (88.6%).

A statistically significant correlation was observed between patients’ depression and age (*P* = .018), type of diagnosis (*P* = .001), NYHA class (*P* = .001), prior hospitalization within the current year (*P* = .004), whether they were current smokers (*P* = .001), whether they consumed alcohol occasionally (*P* = .026), frequency of physical exercise (*P* = .001), and their self-reported ability for symptom management after hospital discharge (*P* = .001). More specifically, patients <60 years of age had a lower rate of depression (66.7%) compared to the other age groups, especially the age group 71–80 years, which recorded a rate of 84.3%. The higher the age group, the higher also the depression rate. Furthermore, higher rates of depression were found in those with a diagnosis of HF decompensation (83.8%) compared to those with chronic HF (54.9%), in those with NYHA class IV (96.7%) compared to those with NYHA III (84.1%) and NYHA I–II (57.4%), those who had prior hospitalization within the current year (81.2%), those with a comorbidity (77.4%), those who did not smoke (82.7%), those who did not consume alcohol (82.6%), those who never performed any type of exercise (86.6%), and those who believed they did not have the ability for symptom management (88.6%).

### Caregivers’ characteristics associated with patients’ Hospital Anxiety and Depression Scale scores

**Table 4** presents the association between caregivers’ characteristics and the presence of anxiety and depression in HF patients. A statistically significant association was observed between HF patients’ anxiety and the relationship with caregivers (*P* = .006), whether caregivers declared added responsibilities among family members (*P* = .041), and whether they felt uncertain about patients’ clinical outcome (*P* = .001). More specifically, patients whose caregivers were their children had a higher percentage of anxiety (89.5%) compared to patients whose caregivers were their spouses (76.4%). Similarly, patients whose caregivers declared added responsibilities among family members and patients whose caregivers felt very uncertain about patients’ clinical outcome had a higher rate of anxiety (83.8% and 89.2%, respectively). A statistically significant correlation was observed between patients’ depression and the relationship with caregivers (*P* = .001), the occupation of caregivers (*P* = .038), whether caregivers declared added responsibilities among family members (*P* = .002), and how uncertain they felt about patients’ clinical outcome (*P* = .001). More specifically, patients whose caregivers were their children had a higher rate of depression (88.6%) compared to patients whose caregivers were their spouses (70.8%). Patients whose caregivers were unemployed had a higher rate of depression (87.8%) compared to patients whose caregivers were employed (73.9%) or retired (72.6%). Similarly, patients whose caregivers declared added responsibilities among family members and patients whose caregivers felt very uncertain about patients’ clinical outcome had a higher rate of depression (82.3% and 91.2%, respectively).

**Table 4:** Caregivers’ Characteristics Associated with Patients’ Anxiety and Depression (HADS Scores)

Caregivers’ Characteristics	Patient Anxiety			Patient Depression		
	No, n (%)	Yes, n (%)	*P* Value	No, n (%)	Yes, n (%)	*P* Value
Relation to patient			**.006**			**.001**
Child	11 (10.5%)	94 (89.5%)		12 (11.4%)	93 (88.6%)	
Husband/wife	46 (23.6%)	149 (76.4%)		57 (29.2%)	138 (70.8%)	
Gender			.672			.439
Male	19 (20.4%)	74 (79.6%)		24 (25.8%)	69 (74.2%)	
Female	38 (18.4%)	169 (81.6%)		45 (21.7%)	162 (78.3%)	
Age (years)			.672			.052
≤40	7 (14.9%)	40 (85.1%)		9 (19.1%)	38 (80.9%)	
41–50	11 (16.9%)	54 (83.1%)		8 (12.3%)	57 (87.7%)	
51–60	15 (25.4%)	44 (74.6%)		23 (39.0%)	36 (61.0%)	
61–70	11 (17.7%)	51 (82.3%)		12 (19.4%)	50 (80.6%)	
71–80	13 (19.4%)	54 (80.6%)		17 (25.4%)	50 (74.6%)	
Education			.697			.934
Primary	10 (15.4%)	55 (84.6%)		16 (24.6%)	49 (75.4%)	
Secondary	24 (20.3%)	94 (79.7%)		27 (22.9%)	91 (77.1%)	
University	23 (19.7%)	94 (80.3%)		26 (22.2%)	91 (77.8%)	
Occupation			.117			**.038**
Unemployed/household	8 (10.8%)	66 (89.2%)		9 (12.2%)	65 (87.8%)	
Employee	33 (21.6%)	120 (78.4%)		40 (26.1%)	113 (73.9%)	
Pensioner	16 (21.9%)	57 (78.1%)		20 (27.4%)	53 (72.6%)	
Frequency of hospital visits			.297			.750
In hospital the whole admission	20 (15.3%)	111 (84.7%)		28 (21.4%)	103 (78.6%)	
Once/twice a day	27 (21.4%)	99 (78.6%)		30 (23.8%)	96 (76.2%)	
Day by day	10 (24.4%)	31 (75.6%)		11 (26.8%)	30 (73.2%)	
Informed about patient’s HF			.682			.718
Very	25 (21.0%)	94 (79.0%)		27 (22.7%)	92 (77.3%)	
Enough	24 (18.6%)	105 (81.4%)		32 (24.8%)	97 (75.2%)	
A little	8 (15.4%)	44 (84.6%)		10 (19.2%)	42 (80.8%)	
Added responsibilities among family members			**.041**			**.002**
Yes	32 (16.2%)	166 (83.8%)		35 (17.7%)	163 (82.3%)	
No	25 (24.5%)	77 (75.5%)		34 (33.3%)	68 (66.7%)	
Uncertain about patients’ clinical outcome			**.001**			**.001**
Very	11 (10.8%)	91 (89.2%)		9 (8.8%)	93 (91.2%)	
Enough	25 (17.7%)	116 (82.3%)		38 (27.0%)	103 (73.0%)	
A little	21 (36.8%)	36 (63.2%)		22 (38.6%)	35 (61.4%)	
Do you neglect your personal life?			.099			.053
Very	11 (12.6%)	76 (87.4%)		11 (12.6%)	76 (87.4%)	
Enough	27 (19.3%)	113 (80.7%)		37 (26.4%)	103 (73.6%)	
A little	19 (26.0%)	54 (74.0%)		21 (28.8%)	52 (71.2%)	
Do caregivers need support?			.772			.145
Very	17 (18.7%)	74 (81.3%)		17 (18.7%)	74 (81.3%)	
Enough	32 (18.3%)	143 (81.7%)		40 (22.9%)	135 (77.1%)	
A little	8 (23.5%)	26 (76.5%)		12 (35.3%)	22 (64.7%)	

*Abbreviations:* HADS, Hospital Anxiety and Depression Scale; HF, heart failure. Bold *P*-values are statistically significant.

### Effect of characteristics on patients’ anxiety and depression

Multiple logistic regression was performed with the patients’ anxiety and depression as dependent variables to estimate the effect of patients’ and caregivers’ characteristics on patients’ anxiety and depression (independent factors) **(Table 5)**.

**Table 5:** Impact of Patients’ and Caregivers’ Characteristics on Patients’ Anxiety and Depression (HADS Scores)

	Patient Anxiety		Patient Depression	
	OR (95% CI)	*P* Value	OR (95% CI)	*P* Value
Age (years)				
≤60	Ref		Ref	
61–70	1.14 (0.40–3.27)	.802	0.83 (0.28–2.43)	.735
71–80	1.70 (0.57–5.07)	.338	1.97 (0.60–6.43)	.263
>80	0.55 (0.14–2.19)	.393	0.88 (0.20–3.92)	.868
Education				
Primary	Ref		—	—
Secondary	0.91 (0.39–2.11)	.825	—	—
University/MSc/PhD	0.45 (0.19–1.10)	.081	—	—
Diagnosis (decompensation vs. chronic HF)	2.26 (1.03–4.99)	**.043**	1.97 (0.82–4.72)	.128
NYHA class				
I–II	Ref		Ref	
III	2.46 (1.07–5.64)	**.034**	2.27 (0.95–5.40)	.064
IV	21.44 (2.41–190.82)	**.006**	3.91 (0.85–17.94)	.080
Prior hospitalization (no vs. yes)	0.94 (0.43–2.02)	.866	0.71 (0.32–1.55)	.389
Smoker (no vs. yes)	1.31 (0.63–2.72)	.465	2.04 (0.91–4.56)	.082
Alcohol consumption (no vs. yes)	—	—	1.29 (0.62–2.70)	.496
Exercise				
Never	Ref		Ref	
1–2 times/week	1.26 (0.51–3.12)	.613	0.78 (0.31–1.99)	.603
>2 times/week	0.66 (0.30–1.46)	.304	0.55 (0.24–1.25)	.152
Able to manage symptoms (no vs. yes)	1.53 (0.71–3.28)	.277	2.27 (1.03–5.00)	.043
Caregiver (spouse vs. child)	0.51 (0.20–1.35)	.177	0.41 (0.14–1.20)	.103
Caregiver’s occupation				
Unemployed/household	—		Ref	
Employee	—		0.51 (0.18–1.48)	.216
Pensioner	—		0.26 (0.08–0.82)	**.022**
Caregivers’ report for added responsibilities among family members (no vs. yes)	0.58 (0.29–1.19)	.140	0.52 (0.25–1.06)	.073
Caregivers’ uncertainty level about patients’ clinical outcome				
Very	Ref		Ref	
Enough	1.39 (0.58–3.31)	.459	0.55 (0.22–1.37)	.198
A little	0.50 (0.19–1.30)	.155	0.55 (0.19–1.58)	.270

Abbreviations: CI, confidence interval; HADS, Hospital Anxiety and Depression Scale; HF, heart failure; NYHA, New York Heart Association; OR, odds ratio; ref, reference category. Bold *P*-values are statistically significant.

Regarding patients’ and caregivers’ characteristics, pa-tients who were diagnosed with HF decompensation were 2.26 times more likely to have anxiety than those diagnosed with chronic HF (odds ratio [OR], 2.26; 95% CI, 1.03–4.99; *P* = .043). Patients with NYHA class III and IV disease were 2.46 and 21.44 times more likely to have anxiety than those with NYHA class I–II disease, respectively (OR, 2.46; 95% CI, 1.07–5.64; *P* = .034) (OR, 21.44; 95% CI, 2.41–190.82; *P* = .006). Finally, patients whose caregivers were pensioners had a 74% lower chance of depression than patients whose caregivers were unemployed (OR, 0.26; 95% CI, 0.08–0.82; *P* = .022).

## Discussion

The present results showed a high prevalence of anxiety and depression. A relevant study in 301 hospitalized HF patients demonstrated anxiety in 41.2% of participants, depression in 58.8%, and both anxiety and depression in 32.6%.^13^ Similarly, a study in a sample of 1260 patients (age, 63.57 ± 13 years) reported anxiety and depression in 52.9% and 32.5%, respectively, and both anxiety and depression in 26.8%.^5^ In Greece, a study in a sample of 190 hospitalized HF patients (46.8% aged >70 years) reported moderate and high levels of anxiety and depression in 57.3% and 41.6%, respectively.^14^ In recognition of the high anxiety and depression prevalence, it is of fundamental importance to systematically assess and treat this burden.^8^

A significant correlation was observed between patients’ anxiety and depression and age, HF diagnosis, NYHA class, prior hospitalization, whether they were current smokers, frequency of physical exercise, and self-reported ability for symptom management after hospital discharge.

According to the literature, several factors are to be held responsible for the anxiety and depression that HF patients experience. Higher rates of anxiety are associated with female sex, living in rural areas, and repeated hospital admissions within the last year. Higher depression rates are related to primary or lower education and normal or low weight.^13^ Women, single patients,^2,15^ those aged <60 years, and those with a low socioeconomic status are more likely to experience depression.^15^ Other factors associated with depression are longer HF duration and living alone. Married HF patients and those with a short HF duration (<1 year) are 59% and 69% less likely to experience major depression, respectively.^14^ Married individuals tend to be more depressed, whereas the unmarried ones tend to exhibit anxiety.^8^ Furthermore, patients with no supportive relatives show a higher probability of experiencing anxiety and depression.^16^ In general terms, factors associated with anxiety and depression are: (1) patients’ demographic characteristics, such as advanced age, female sex, and low socioeconomic status; (2) patients’ clinical characteristics, such as disease severity, comorbidities, and medication use (β-blockers); and (3) psychosocial factors, such as social support, coping mechanisms, and personality traits.^1^

Patients <60 years of age had lower rates of anxiety and depression. A contradictory option is that younger patients experience greater psychological distress due to the challenge of reconciling a progressive physical decline with expectations for a long, and productive life. Conversely, despite higher morbidity and mortality, the elderly report better quality of life and greater optimism.^17,18^

The results of the current study indicating that higher rates of anxiety and depression were present in patients with NYHA class IV disease are in line with other studies. The severity of depression increases with NYHA class but not anxiety.^8^ Depression prevalence increases with the severity of symptoms, from 11% in patients with NYHA class I disease to 20% with NYHA II, 38% with NYHA III, and 42% with NYHA IV disease, respectively.^19^ The importance of NYHA class in the long-term treatment of HF needs to be emphasized, as it is predictive of cardiopulmonary function, physical status, quality of life, and clinical outcomes, including mortality.^8^ Moreover, in hospitalized patients, an increase in NYHA class was associated with a 3.4-fold greater risk of cardiac events.^20^ Furthermore, depression, NYHA III/IV, diabetes mellitus, chronic kidney disease, muscle weakness, and slow gait are risk factors for hospital admission.^21^

Perhaps of greater concern is the current finding that HF patients who never performed any type of exercise experienced anxiety and depression. Decreased exercise capacity is prognostic for disease progression.^22,23^ In depressed patients, regular activity decreases HF risk, whereas discontinuation of regular activity increases HF risk.^23^ Also, of importance is the acknowledgment that the benefits of exercise span the entirety of the disease trajectory. Specifically, exercise has a protective benefit in preventing HF (primary prevention), but when HF is established, exercise provides benefits to HF patients (secondary prevention).^22^

HF patients who reported no ability for symptom management after hospital discharge experienced anxiety and depression. Self-care is a crucial aspect of HF treatment and consists of three dimensions. The first dimension (self-care maintenance) includes behaviors such as treatment adherence, the second (self-care monitoring) includes recognition of symptoms, and the third (self-care management) is the response to deteriorating symptoms, which also involves decision-making. In self-management, when a symptom occurs or deteriorates (edema, dyspnea), the HF patient is asked not only to recognize it but also to take actions (receive additional diuretics) and afterward to evaluate whether this choice was effective.^24^ Unfortunately, anxiety and depression are inversely associated with self-care in HF patients.^25^ Reduced self-care capacity, hopelessness, despair, and diminished social relationships are only some of the effects of depressive symptoms.^26^ Inadequate self-care and low social support increase hospitalization risk.^27,28^ In contrast, better self-care is associated with improvements in depression during 6-month follow-up and reductions in anxiety scores at 6 weeks.^29^ One’s self-care ability is also influenced by factors related either to the health care system of each country, such as access to health services, or to health care professionals, such as failure to comprehend HF patients’ needs and afterward to integrate them into the framework of care.^24^ More strikingly, the progressive nature of HF increases patients’ doubts about the usefulness of self-care, thus reducing their motivation for self-care.^30^ An intervention that enhances self-care is the participation of a caregiver (spouse) in the treatment regimen. Notably, caregiver activities include symptom monitoring, assistance with medication and dietary restrictions, inclusion in social activities, emotional support, and end-of-life care.^31,32^ Education and provision of elaborate information to cardiac patients and caregivers should be an ongoing effort.^33,34^

A considerable finding showed higher percentages of anxiety and depression among HF patients whose caregivers declared uncertainty about patients’ clinical outcome and added responsibilities among family members. Possibly, HF patients perceive that they impose a heavy burden on caregivers, experiencing anxiety and depression as a result. According to the “emotional contagion” theory, the burden is spread to others, particularly when two individuals are in a close interpersonal relationship.^35^ Interestingly, caregivers of HF patients who are also described as “invisible victims” struggle with disease deterioration, poor prognosis, and demanding daily tasks, which may last for several years.^36^

What is more intriguing is that data demonstrated anxiety and depression among HF patients whose caregivers were their children. Similarly, a relevant study in China reported higher anxiety scores in HF patients when caregivers were their children and higher depression scores when caregivers were their spouses or other close relatives. Based on the “intergenerational trauma,” the psychological trauma is transmitted from survivor to descendants.^35^ Provided that child caregivers are of a younger age, it is easily understood that they are offered wider support opportunities such as via digital services or mobile applications specific to the patient’s restrictions.^37^ Minimizing children’s caregiving burden is a way to alleviate patients’ anxiety and depression.

Furthermore, patients whose caregivers were unemployed had a higher rate of depression. Economic stability is a major component in meeting survival needs, which is also fundamental to positive mental health and well-being.^38^ Depression of HF patients is partially explained by the absence of security when caregivers are unemployed. On the contrary, unemployment determines the choice of a child to provide informal care to a loved one in the family. An increase in the availability of informal care indicates an increase in unemployment rates.^39^

In clinical practice, health care professionals provide care to HF patients from diagnosis to ongoing treatment, with the ultimate goal of improving their outcomes. Frequently, they pay more attention to disease management and complex therapeutic regimens, thus focusing less on patients’ mental health. According to recommendations from the Heart Failure Association of the European Society of Cardiology (2021), it is of crucial importance to screen for anxiety and depression by self-reported questionnaires and involve family as well as to consider referral for psychiatric/psychological consultation.^40^ As previously mentioned, the benefit arising from systematically exploring and alleviating mental burden in HF treatment is translated to fewer hospital readmissions, minimized health care costs, and decreased morbidity or mortality.^6,7^ Therefore, the present results may increase awareness among clinicians to identify patients at risk or those with established mental burdens. The major criticality of this study is to identify factors associated with the mental health of hospitalized HF patients along with caregivers’ characteristics, thus elucidating strategies to alleviate this burden.

### Study limitations

In the present cross-sectional study, there was no evidence of a causal relationship between patients’ and caregivers’ characteristics and patients’ anxiety and depression. The convenience sampling method is not representative of all HF hospitalized patients in Greece, thus limiting the generalizability of the results. Furthermore, there were no other measurements that may permit exploration of all possible changes in factors associated with anxiety and depression.

## Conclusions

A higher rate of anxiety and depression was observed in patients with HF decompensation, those with NYHA class IV disease, those with a prior hospitalization within the current year, those who did not smoke, those who never performed any type of exercise, and those who declared having no ability for symptom management after hospital discharge. Also, the higher the age group, the greater the anxiety and depression rates. Meanwhile, a higher rate of anxiety was observed among HF patients with only primary education, while a higher rate of depression was reported among patients who did not consume alcohol.

A higher percentage of anxiety and depression was found in HF patients whose caregivers were their children, whose caregivers declared added responsibilities among family members, and whose caregivers felt very uncertain about patients’ clinical outcome. Patients whose caregivers were unemployed had a higher rate of depression.

### Future perspectives in clinical practice

In terms of future expectations, it is essential to evaluate anxiety and depression among HF patients along with caregivers’ characteristics. From a clinical practice perspective, health care professionals need to measure anxiety and depression at hospital admission in order to screen HF patients with an already established emotional burden or those at high risk. The next step is to offer a multidisciplinary approach to HF patients with anxiety and depression, including consultation with a psychologist/psychiatrist. Finally, before hospital discharge, a second measurement of anxiety and depression is essential to record anticipated improvements and schedule a prompt follow-up and subsequent monitoring. All the aforementioned actions should include assessment of the caregivers’ profile/characteristics, as they were shown to affect patients’ mental health.
